# PReS-FINAL-2206: Clinical experiences in patients with chronic recurrent multifocal osteomyelitis (CRMO)

**DOI:** 10.1186/1546-0096-11-S2-P196

**Published:** 2013-12-05

**Authors:** B Sozeri, B Yildiz, M Sezak, M Argin

**Affiliations:** 1 Pediatric Rheumatology, Ege University, Izmir, Turkey; 2 Pediatric Infectious Disease, Ege University, Izmir, Turkey; 3 Pathology, Ege University, Izmir, Turkey; 4 Radiology, Ege University, Izmir, Turkey

## Introduction

Chronic recurrent multifocal osteomyelitis (CRMO) is a form of chronic nonbacterial osteitis (CNO) characterized by one or more lytic bone lesions with no identifiable cause. (CRMO) is a rare (prevalence less than 1/1,000,000) auto-inflammatory disorder, characterized by relapsing and remitting episodes of pain related to the presence of foci of sterile bone inflammation.

## Objectives

We describe the clinical and laboratory features and treatment of a cohort of children with CRMO.

## Methods

We retrospectively reviewed clinical, pathological and radiological data of children with CRMO at single tertiary pediatric center from Turkey. The diagnosis of CRMO was based on evidence of recurrent osteomyelitis with radiographic evidence of chronic osteomyelitis involving at least two sites in the absence of infectious cause in a child less than 14 years old.

## Results

Six patients were assessed (34 females and 6 males) with a median age at diagnosis of 9,5 yrs (range 5-16). Median number of initial bony lesions was 2 at onset and 3.5 over disease course. Median time since diagnosis was 1.5 yrs) and median duration of active disease 0.7 yrs. Two patients had active disease at follow-up and continued to have pain. Two patients were treated with steroid, colchicine and sulphasalazine. Two patients had methotrexate, 1 had colchicine while 1 had nonsteroidal anti-inflammatory drug.

## Conclusion

This report indicates that CRMO may be overlooked in our community. Early diagnosis and treatment are required to avoid potential complications.

## Disclosure of interest

None declared.

